# Bioinspired Spatio-Temporal Cooperative Path Planning for Heterogeneous UAVs Driven by Bi-Level Games: An SSA-MPC Fusion Approach

**DOI:** 10.3390/biomimetics11040286

**Published:** 2026-04-21

**Authors:** Yaowei Yu, Meilong Le

**Affiliations:** Department of Transportation, College of Civil Aviation, Nanjing University of Aeronautics and Astronautics, Nanjing 211106, China

**Keywords:** biomimetic robotics, bioinspired behavioral algorithm, Sparrow Search Algorithm, heterogeneous UAVs, event-triggered MPC, cooperative path planning

## Abstract

Collaborative operation of heterogeneous UAV swarms in dense urban environments remains challenging because right-of-way allocation is often rigid, frequent replanning consumes considerable onboard computation, and paths obtained by purely mathematical optimization may not be easy to execute under real dynamic constraints. This paper presents a physics-informed, event-triggered path planning and control framework, termed Physics-Informed SSA-MPC. Its global search layer is built on the Sparrow Search Algorithm (SSA), whose search mechanism originates from sparrow foraging and anti-predatory behaviors. On this basis, the method combines an event-triggered Stackelberg game for airspace coordination, a physically constrained SSA for global path generation, and an event-triggered MPC for local replanning. Battery State of Health (SoH) is incorporated into the adaptive search process, while Lévy-flight updates are limited by the maximum available acceleration to avoid infeasible path mutations. Local replanning is activated only when predicted safety ellipsoids overlap or tracking errors exceed prescribed thresholds, which helps reduce redundant computation. Simulations in a digital twin of Lujiazui, Shanghai, show that the proposed method shortens path length by 3.3% to 14.9%, reduces obstacle-avoidance latency to 45 ms, and achieves a 100% engineering feasibility rate.

## 1. Introduction

Heterogeneous UAV swarms are being used more often in low-altitude tasks such as urban logistics and emergency response [1,2]. In dense urban areas, they must deal with building occlusion, canyon wind disturbances, and moving obstacles at the same time. The platforms in one swarm may also differ a lot, from heavy-lift logistics UAVs to more agile inspection vehicles, so their dynamic capabilities and mission priorities are usually not the same. Under these conditions, path planning becomes a high-dimensional and non-convex problem [3,4]. The main difficulty is not only how to find good paths, but also how to keep them physically feasible and computationally manageable for onboard implementation.

While theoretical advancements in UAV path planning are substantial, three critical engineering bottlenecks persist during actual low-altitude urban flight.

First, conventional airspace resource allocation remains overly rigid; this static approach offers minimal fault tolerance. Most existing coordination models rely on static game logic, pre-defining leader–follower roles before takeoff. Such open-loop mechanisms lack necessary feedback, making them ill-equipped to handle sudden disturbances [5,6,7,8]. If a follower drone experiences power degradation due to strong gusts or battery aging, the fixed avoidance rules may lead to trajectory divergence, potentially triggering a chain of collisions [8].

Second, paths generated by purely mathematical heuristic optimization often lack physical flyability. Conventional intelligent search algorithms (e.g., PSO, SSA) frequently utilize stochastic step updates to escape local optima. This often results in sharp turns or significant spatial jumps that violate the dynamic constraints of multi-rotor UAVs. Executing these unfeasible commands can cause motor overload or flight control system failure [9,10,11,12]. More fundamentally, neglecting the State of Health (SoH) of batteries causes a severe disconnect between scheduling strategies and the drones’ actual performance [13,14].

Third, high-frequency dynamic obstacle avoidance mechanisms drain excessive computational resources. Currently, Model Predictive Control (MPC) is the standard for online collision avoidance [15,16]. However, traditional MPC employs fixed-frequency receding horizon optimization, performing redundant calculations even in safe environments. Given the limited onboard capacity of small UAVs, this redundancy induces command latency, potentially leading to the failure of avoidance systems in highly dynamic settings [17,18,19].

To address these issues, this study proposes a Physics-Informed, Event-Triggered Game-Control integrated path planning method (Physics-Informed SSA-MPC). The framework combines an improved Sparrow Search Algorithm for global path generation, an event-triggered Stackelberg game for airspace coordination, and an event-triggered MPC for local replanning. The global search layer is based on the Sparrow Search Algorithm (SSA), whose search mechanism is inspired by sparrow foraging and anti-predatory behaviors, and is extended here to heterogeneous UAV coordination and control in complex urban environments. The primary contributions of this work are summarized as follows:

1. An event-triggered game–theoretic coordination architecture with dynamic role switching is designed. By replacing static right-of-way allocation with a feedback-enabled Stackelberg game, the proposed method links low-level state monitoring with high-level airspace coordination. When a UAV’s tracking error approaches the safety boundary, its game priority is adaptively increased, allowing the system to reallocate spatiotemporal resources and reduce conflict escalation under disturbances.
2. A physically constrained global search strategy is developed based on an improved SSA. Battery State of Health (SoH) is incorporated into adaptive search weighting, and maximum acceleration limits are imposed on Lévy-flight step updates. This design improves global exploration while avoiding dynamically infeasible path mutations.
3. An event-triggered local replanning framework is established to reduce redundant computation. Instead of using fixed-frequency optimization, the MPC module is activated only when predicted safety ellipsoids overlap or tracking errors exceed predefined thresholds. This mechanism improves response efficiency while lowering unnecessary onboard computational burden during safe flight periods.
4. A rigorous theoretical framework for closed-loop stability is constructed. Utilizing Lyapunov stability theory, we prove that the proposed event-triggered MPC system achieves Input-to-State Stability (ISS) under bounded wind field disturbances, providing solid mathematical grounding for urban low-altitude applications.

The remainder of this paper is organized as follows: Section 2 reviews related work; Section 3 establishes the heterogeneous UAV dynamics and complex urban environment models; Section 4 details the dual-layer coordination architecture and the Physics-Informed SSA-MPC mechanism; Section 5 presents the convergence and stability derivations; Section 6 conducts extensive simulation benchmarks and experiments within a digital twin of Shanghai’s Lujiazui district; Section 7 concludes the study.

## 2. Related Work

Research related to this study mainly falls into three lines: heterogeneous multi-UAV path planning, improvements to the Sparrow Search Algorithm (SSA), and the use of Model Predictive Control (MPC) in UAV systems. In much of the existing literature, these topics are often discussed separately. Some studies emphasize global search efficiency, some focus on coordination among multiple vehicles, and others concentrate on local tracking or obstacle avoidance. For the problem considered here, these aspects are closely coupled. The following review therefore focuses on the main progress in each line of work and the issues that still limit practical deployment in heterogeneous UAV swarms.

### 2.1. Research on Heterogeneous Multi-UAV Path Planning

The main goal of heterogeneous multi-UAV path planning is to generate trajectories that remain safe, efficient, and coordinated for platforms with different dynamic capabilities and task priorities. Earlier North American work has also addressed heterogeneous UAV cooperation through coupled task assignment and reactive motion planning, showing that coordination and path generation are naturally intertwined in multi-platform missions [20]. This becomes much harder in urban environments, where buildings, narrow corridors, wind disturbances, and moving obstacles interact with one another. Existing studies usually approach the problem from three directions: classical graph-based search, swarm intelligence optimization, and MPC-based planning or control frameworks [17,21,22]. Each direction has clear strengths, but none fully resolves the coupled spatial, temporal, and dynamic constraints found in dense low-altitude urban operations.

Graph-based algorithms, such as A*, Dijkstra, and RRT, represent the classical cornerstone of UAV navigation, particularly in static, single-agent scenarios [21,22,23]. For instance, scholars have integrated radar threat estimation and multi-layer variable-step search strategies into the A* framework to balance waypoint precision with search efficiency [24]. Others have utilized A* to output obstacle density metrics, facilitating subsequent trajectory refinement [25]. Furthermore, multi-objective guidance and dynamic safety constraint mechanisms have been incorporated to generate collision-free paths for multi-UAV systems, outperforming various RRT variants in both length and smoothness [26]. However, these methods struggle with the “dimensionality explosion” inherent in heterogeneous swarms. The exponentially increasing computational complexity and the inability to directly handle spatio-temporal coordination constraints often preclude the discovery of globally optimal solutions.

Swarm Intelligence (SI) algorithms have emerged as the mainstream solution for heterogeneous planning due to their robust distributed search and global optimization capabilities [27]. Recent studies have proposed adaptive multi-population whale optimization to balance exploration and exploitation, though these often neglect the heterogeneity of the swarm [28]. While gray wolf optimization variants have addressed multi-UAV coordination, they suffer from sluggish convergence in high-dimensional spaces [29]. To tackle formation planning, the Obstacle Avoidance Empowered Consensus (OAEC) algorithm was coupled with Multi-step Particle Swarm Optimization (MPSO); however, this framework lacks resiliency, requiring full re-computation upon single-agent failure or the emergence of new threats [30]. For time-constrained heterogeneous tasks, the Improved Raid-Siege WPA (MRS-WPA) introduced a composite walk-fly mechanism—utilizing triangular wandering and Lévy flights—to escape local optima [31]. Despite enhancing precision, it remains vulnerable to stochastic dynamic obstacles. Hybrid approaches combining deep learning (CNN/LSTM) with multi-objective PSO have also been explored to predict and optimize trajectories for high-speed stability [32]. A path prediction model was established using a convolutional neural network (CNN) and a long short-term memory (LSTM) network to estimate trajectory points. Multi-objective particle swarm optimization (MOPSO) was used to optimize and generate trajectories, ensuring good flight stability and safety while maintaining high-speed flight. However, trajectory prediction for dynamic obstacles and real-time obstacle avoidance were not considered. More recently, learning-based methods such as deep reinforcement learning and large AI model deployment have also been explored for aircraft path planning and low-altitude autonomous decision making [33,34]. These studies show clear potential for adaptive policy learning in complex environments. At the same time, bringing such methods into heterogeneous UAV swarms with strict physical constraints, hard safety margins, and limited onboard resources is still not straightforward. Nevertheless, a persistent gap remains: existing SI algorithms generally exhibit poor coordination synergy and insufficient adaptability to highly dynamic environments.

### 2.2. Improvements in Sparrow Search Algorithm (SSA)

The Sparrow Search Algorithm (SSA), proposed in 2020, is a swarm intelligence method derived from sparrow foraging and anti-predatory behaviors. Because its update rules are simple and its convergence is often fast, it has been used in many optimization and path planning problems [35]. In UAV-related studies, most improvements to SSA have focused on the search process itself. Some works adjust weights or add mutation-based perturbations to help the algorithm escape local optima [36]. Others use chaotic initialization, opposition-based learning, or Lévy-flight updates to improve exploration and convergence stability [10,37]. There are also studies that combine SSA with PSO or bio-inspired neural networks to improve path quality in specific scenarios [11,38]. Dynamic step-size schemes and other search operators have also been introduced to improve convergence speed and path quality [39]. Even so, one issue remains quite common. These changes usually improve the algorithm in a mathematical sense, but they do not directly bring the physical state of the UAV into the update process. Battery health, actuator capability, and acceleration limits are still rarely embedded in the search loop itself. Recent European work on constrained or time-optimal UAV trajectory generation has shown that explicit acceleration, velocity, thrust, or replanning constraints are crucial for turning numerically good trajectories into trajectories that remain executable in practice [40,41]. Because of that, a path may look better in computation while still being difficult to execute smoothly on a real UAV.

### 2.3. Model Predictive Control

Leveraging the advantages of receding horizon optimization and explicit constraint handling, Model Predictive Control (MPC) has emerged as a predominant methodology for dynamic trajectory tracking and obstacle avoidance in UAV systems [17,42]. While existing literature has demonstrated fixed-wing path following based on linear MPC, other studies have successfully addressed dynamic obstacle avoidance for multi-rotor UAVs through Nonlinear MPC (NMPC) [43,44].

Current research trajectories for MPC applications in the UAV domain are well-defined and robust: starting from local control problems of single UAVs, significant milestones have been achieved in both trajectory tracking precision and real-time collision avoidance. Given that standard MPC must predict future system behavior [45], it inherently entails high computational complexity [46]. Representative work from North American groups has addressed this issue through efficient or constrained MPC formulations for quadrotor tracking and path following, with explicit attention to onboard implementability and stability [47,48]. To mitigate this, researchers introduced Efficient Model Predictive Control (EMPC), which ingeniously utilizes linear internal models to simplify prediction points, thereby substantially alleviating the computational burden [47]. Concurrently, a linear constrained MPC approach was proposed in [48], employing Laguerre functions to estimate control input sequences, effectively reducing computational requirements while maintaining performance. Complementing these advancements, a distributed MPC algorithm based on swarm intelligence in [49] incorporates an event-triggered strategy to directly prune the computational overhead of decentralized frameworks.

Nevertheless, it is pertinent to note that the performance of methods in [47,48] may deteriorate under external disturbances such as wind fields. To counter these environmental perturbations, a robust time-varying MPC (TCMPC) was developed [45], which demonstrates superior stability in the translational and attitudinal control of quadrotors. More recent and sophisticated integration techniques involve combining Gaussian Processes (GP) or $L_{1}$ adaptive theory with MPC to compensate for residual uncertain internal dynamics, thereby significantly enhancing path-following accuracy in complex environments [50,51].

In the realm of multi-agent coordination, Distributed MPC (DMPC) based on Nash optimization has yielded prominent experimental results in collaborative search missions across multiple platforms [52]. By integrating genetic programming with DMPC, ref. [53] autonomously generated collaborative search strategies that satisfy multiple constraints, markedly improving temporal efficiency for dynamic target acquisition and adaptability to intricate environments. Notably, a Distributed Stochastic MPC (DSMPC) algorithm was proposed in [54] specifically for the coordinated control of heterogeneous UAV swarms subject to external disturbances, incorporating reasonable obstacle avoidance constraints. The pivotal concept involves optimizing only a single UAV at each time step, allowing the collective coordination to be elegantly decoupled into several local sub-problems with chance constraints. Finally, an integrated “global planning–local tracking” control methodology, combining linearized models with NMPC in [55], offers a clear and insightful paradigm for real-time dynamic obstacle avoidance in multi-rotor systems.

Despite the evident efficacy of these enhanced MPC strategies, two critical challenges remain unresolved when scaling to heterogeneous multi-UAV swarm scenarios. First, the redundancy of periodic computation in obstacle-free environments has not been addressed, leading to inefficient utilization of computational resources. Second, relying solely on MPC for local optimization risks entrapping the system in local optima, thereby failing to guarantee global trajectory optimality. Consequently, there is a compelling need to design more sophisticated MPC mechanisms tailored for practical operational contexts.

## 3. Heterogeneous UAV Dynamics and Environmental Constraint Models

This section constructs a second-order dynamic model for heterogeneous multi-rotor UAVs, quantifies urban low-altitude environmental constraints and collision avoidance boundaries, and establishes a multi-objective optimization cost function. These elements provide a comprehensive modeling foundation for subsequent algorithm design. The model is developed within a Cartesian coordinate system, encompassing a three-dimensional operational space defined by x-, y- and z-axes.

### 3.1. Second-Order Dynamic Model for Heterogeneous Multi-Rotors

The focus of this study is a swarm of heterogeneous multi-rotor UAVs. The second-order model used in this paper is aimed at the planning and coordination layer, not at full flight dynamics or attitude-level control. It is suitable for low-altitude multi-rotor tasks where the inner-loop controller can follow acceleration commands on a faster time scale. In this setting, the model is detailed enough for cooperative path planning, safety analysis, and receding horizon replanning, while keeping the problem simpler than a full nonlinear aerodynamic model.

The set of heterogeneous attributes for the ith UAV is defined as shown in Equation (1):

(1) $$A_{i}=\{R_{i},m_{i},v_{\max}^{i},a_{\max}^{i}\}$$

where $R_{i}$ denotes the equivalent fuselage radius, $m_{i}$ represents the takeoff mass, $v_{\max}^{i}$ is the maximum level flight speed, and $m_{i}$ signifies the maximum maneuverable acceleration.

The second-order kinematic state-space equations for the UAV in 3D space are expressed as:

(2) $$\left\{\begin{array}{l} p_{i}(t+\Delta t)=p_{i}(t)+v_{i}(t)\Delta t+\frac{1}{2}a_{i}(t)\Delta t^{2}\\ v_{i}(t+\Delta t)=v_{i}(t)+a_{i}(t)\Delta t \end{array}\right.$$

where $p_{i}(t)\in {\mathbb{R}}^{3}$ is the position vector of the UAV at time t, $v_{i}(t)\in {\mathbb{R}}^{3}$ is the velocity vector, $a_{i}(t)\in {\mathbb{R}}^{3}$ is the acceleration vector, and Δt denotes the control period.

The control inputs of the UAV must adhere to hard physical boundary constraints, which are directly embedded into both the global path planning and the MPC local replanning processes:

(3) $${\left\|v_{i}(t)\right\|}_{2}\leq v_{\max}^{i},{\left\|a_{i}(t)\right\|}_{2}\leq a_{\max}^{i}$$

### 3.2. Urban Low-Altitude Environmental Constraint Models

#### 3.2.1. Static Environmental Constraints

The urban static environment primarily comprises buildings, No-Fly Zones (NFZs), and Limited-Flight Zones (LFZs), which are mathematically described using cubic models. The model for the k-th building is given by:

(4) $$\Omega_{b,k}=\left\{(x,y,z)\left|x\in [x_{s,k},x_{e,k}],y\in [y_{s,k},y_{e,k}],z\in [0,h_{b,k}]\right.\right\}$$

where $x_{s,k},x_{e,k}$ and $y_{s,k},y_{e,k}$ define the start and end coordinates of the building along the x-axis and y-axis, respectively, and $h_{b,k}$ is the building height.

The models for NFZs and LFZs are defined as follows:

(5) $$\Omega_{nf,j}=\left\{(x,y,z)\left|x\in [x_{nf,s,j},x_{nf,e,j}],y\in [y_{nf,s,j},y_{nf,e,j}],z\in [0,h_{nf,j}]\right.\right\}$$

(6) $$\Omega_{rf,l}=\left\{(x,y,z)\left|x\in [x_{rf,s,l},x_{rf,e,l}],y\in [y_{rf,s,l},y_{rf,e,l}],z\in [0,h_{rf,\min,l}]\right.\right\}$$

where $h_{nf,j}$ is the maximum height of the NFZ, and $h_{rf,\min,l}$ is the minimum altitude limit of the LFZ. The UAV path points must satisfy $P_{i,s}\notin \Omega_{nf,j}$; additionally, if $P_{i,s}\in \Omega_{rf,l}$, then $z_{i,s}\geq h_{rf,\min,l}$ must be met.

#### 3.2.2. Dynamic Environmental Disturbance Models

Dynamic disturbances in urban low-altitude airspace mainly include canyon wind fields and dynamic obstacles, which directly impact flight safety and path planning efficacy.

1. Canyon Wind Field Disturbance Model

Urban street canyons generate stochastic wind fields. The wind field vector at position p=(x,y,z) at time t is expressed as:

(7) $$v_{w}(p,t)={\bar{v}}_{w}(p)+{\tilde{v}}_{w}(p,t)$$

where ${\bar{v}}_{w}(p)$ is the average wind velocity vector at that location, calibrated via an urban low-altitude wind field measurement database. ${\tilde{v}}_{w}(p,t)$ represents the random gust velocity vector, which follows a 3D Gaussian distribution with a zero mean $0_{3\times 1}$ and covariance matrix $\Sigma_{wind}$, denoted as ${\tilde{v}}_{w}(p,t)\sim N(0,\Sigma_{wind})$. This mean-wind-plus-Gaussian-gust model is used here as a simplified disturbance representation for online planning and control. It does not resolve all fine-scale aerodynamic effects around complex urban structures, but it is sufficient for evaluating the sensitivity of the proposed framework to bounded wind variations within the current simulation setting.

2. Dynamic Obstacle Motion Model

Dynamic obstacles, such as other mobile UAVs or birds, are modeled as moving spheres. The position of the mth dynamic obstacle at time t is $p_{obs,m}(t)$, with a safety radius $R_{obs,m}$, described by:

(8) $$\Omega_{obs,m}(t)=\left\{(x,y,z)\left|\|p-p_{obs,m}(t){\|}_{2}\leq R_{obs,m}\right.\right\}$$

This study employs a Constant Acceleration (CA) model to describe the motion characteristics of dynamic obstacles. Kalman filtering is utilized to estimate the obstacle’s position, velocity, and acceleration in real time, providing a predictive basis for MPC receding horizon optimization. The CA model with Kalman filtering is adopted here as a lightweight short-horizon predictor for local replanning. This assumption is reasonable for online prediction over a limited horizon, although its accuracy may decrease when obstacles perform highly aggressive maneuvers.

### 3.3. Anisotropic Dynamic Safety Ellipsoid

To enhance the utilization of scarce urban airspace, this study discards traditional static circular collision boundaries in favor of an anisotropic dynamic safety ellipsoid model, predicated on heterogeneous UAV performance variations and real-time wind conditions.

The safety ellipsoid is centered at the UAV’s real-time position $p_{i}(t)$. The braking major axis $r_{long}^{i}$ along the velocity direction and the minor axis $r_{lat}^{i}$ perpendicular to the velocity direction are defined as:

(9) $$r_{long}^{i}=R_{i}+\frac{\|v_{i}(t)-v_{w}(p_{i}(t),t){\|}_{2}^{2}}{2a_{\max}^{i}}$$

(10) $$r_{lat}^{i}=R_{i}+\Delta_{wind}$$

where $\Delta_{wind}$ represents the crosswind yaw safety margin, set to a default value of 2 m in this study.

Based on this model, the collision avoidance constraint for the heterogeneous swarm is transformed such that, at any given time, the dynamic safety ellipsoids of any two UAVs do not algebraically overlap:

(11) $$\frac{{\left(x_{j}-x_{i}\right)}^{2}}{{\left(r_{long}^{i}+r_{long}^{j}\right)}^{2}}+\frac{{\left(y_{j}-y_{i}\right)}^{2}}{{\left(r_{lat}^{i}+r_{lat}^{j}\right)}^{2}}+\frac{{\left(z_{j}-z_{i}\right)}^{2}}{{\left(r_{lat}^{i}+r_{lat}^{j}\right)}^{2}}>1,\forall i\neq j$$

This constraint fundamentally ensures the spatio-temporal collision safety of heterogeneous UAVs while improving airspace utilization by over 30% compared to static circular boundaries.

### 3.4. Multi-Objective Optimization Cost Function

A multi-objective optimization function is constructed, comprising individual UAV costs and multi-UAV coordination costs. A lower objective function value indicates superior path planning performance:

(12) $$J=\sum_{i=1}^{N}\omega_{i}J_{i,ind}+\gamma J_{coop}$$

where N is the number of UAVs, $J_{i,ind}$ is the individual cost of the i-th UAV, $J_{coop}$ is the multi-agent coordination cost, $\omega_{i}$ is the individual cost weight (satisfying $\sum_{i=1}^{N}\omega_{i}=1$), and γ is the coordination cost weight.

#### 3.4.1. Individual Cost Function

The individual cost function synthesizes the core operational requirements of UAV flight, expressed as:

(13) $$J_{i,ind}=\omega_{1}J_{i,energy}+\omega_{2}J_{i,smooth}+\omega_{3}J_{i,risk}+\omega_{4}J_{i,air}$$

where $\omega_{1},\omega_{2},\omega_{3},\omega_{4}$ are weighting coefficients satisfying $\omega_{1}+\omega_{2}+\omega_{3}+\omega_{4}=1$. The sub-costs are calculated as follows:

Flight Energy Cost $J_{i,energy}$: This is normalized based on the total energy consumption, integrating the effects of propulsion degradation and wind disturbances. A lower value signifies higher energy efficiency:

(14) $$J_{i,energy}=\frac{E_{i,actual}}{E_{i,max}\cdot \eta_{m,i}(t)}$$

where $E_{i,actual}$ is the actual total energy consumption, $E_{i,max}$ is the theoretical maximum energy, and $\eta_{m,i}(t)$ is the efficiency degradation coefficient of the propulsion system. The efficiency $\eta_{m,i}(t)$ is dynamically updated based on the State-of-Health (SoH) metrics derived in [56].

Path Smoothness Cost $J_{i,smooth}$: Calculated based on the average curvature of the path. Lower values indicate higher smoothness and reduced energy expenditure for attitude adjustments:

(15) $$J_{i,smooth}=\frac{1}{M}\sum_{s=1}^{M}|\kappa_{i,s}|$$

where M is the number of path points and $\kappa_{i,s}$ is the curvature of the i-th UAV’s path at the s-th point.

Obstacle Avoidance Risk Cost $J_{i,risk}$: Evaluates collision risks with both static and dynamic obstacles. Lower values reflect lower risk:

(16) $$J_{i,risk}=\sum_{k=1}^{N_{obs}}\lambda_{risk}\cdot \exp\left(-\frac{d_{i,k,\min}}{d_{safe}+\epsilon }\right)$$

where $N_{obs}$ is the total number of obstacles, $d_{i,k,\min}$ is the minimum distance between the path and the k-th obstacle, $d_{safe}=5\;m$ is the safety distance, and $\lambda_{risk}$ is the risk coefficient. ϵ is a small positive number used to prevent the occurrence of numerical calculation singularities when the distance is zero.

Airspace Violation Cost $J_{i,air}$: Assesses the risk of violating airspace regulations. Costs escalate sharply upon entering NFZs or unauthorized LFZs:

(17) $$J_{i,air}=\lambda_{air}\cdot (N_{nf,i}+N_{rf,i})$$

where $\lambda_{air}$ is the violation penalty coefficient, $N_{nf,i}$ is the count of NFZ entries, and $N_{rf,i}$ is the count of LFZ violations.

#### 3.4.2. Multi-UAV Coordination Cost Function

The coordination cost function integrates the collaborative constraints of multi-UAV flight, expressed as:

(18) $$J_{coop}=\gamma_{1}J_{collision}+\gamma_{2}J_{comm}+\gamma_{3}J_{time}+\gamma_{4}J_{airspace}$$

where $\gamma_{1},\gamma_{2},\gamma_{3},\gamma_{4}$ are weighting coefficients satisfying $\gamma_{1}+\gamma_{2}+\gamma_{3}+\gamma_{4}=1$. The sub-costs are as follows:

Multi-UAV Collision Risk Cost $J_{collision}$: Evaluates inter-UAV collision risks, with costs increasing drastically when safety ellipsoids overlap:

(19) $$J_{collision}=\sum_{t}\sum_{i=1}^{N}\sum_{j=i+1}^{N}\lambda_{collision}\cdot \exp\left(-\frac{{\left\|p_{i}(t)-p_{j}(t)\right\|}_{2}}{r_{long}^{i}+r_{long}^{j}+\epsilon }\right)$$

where $\lambda_{collision}$ is the collision risk coefficient.

Communication Interruption Cost $J_{comm}$: Assesses the communication stability of the swarm, imposing penalties if communication distances are exceeded:

(20) $$J_{comm}=\lambda_{comm}\cdot N_{comm,break}$$

where $\lambda_{comm}$ is the penalty coefficient and $N_{comm,break}$ is the frequency of communication loss. Here, communication interruption is described in a simple planning-layer form. When the distance between cooperative UAVs exceeds a preset communication radius, the link is treated as unreliable and a communication loss penalty is added.

Temporal Coordination Deviation Cost $J_{time}$: Evaluates the coordination error in arrival times. Lower values represent higher coordination precision:

(21) $$J_{time}=\frac{1}{N}\sum_{i=1}^{N}|t_{i,arr}-t_{i,target}|$$

where $t_{i,arr}$ and $t_{i,target}$ are the actual and target arrival times, respectively. This term measures the difference between the actual arrival time and the desired coordination time at shared task points. It is used to keep heterogeneous UAVs temporally aligned during cooperative missions.

Airspace Resource Utilization Cost $J_{airspace}$: Evaluates the balance of airspace resource allocation. Lower values signify higher efficiency:

(22) $$J_{airspace}=\frac{1}{T}\sum_{t=1}^{T}\sigma_{N}(t)$$

where $\sigma_{N}(t)$ is the standard deviation of the UAV positions at time t. The position standard deviation is used to describe how evenly the swarm occupies the available airspace. Smaller values indicate a more balanced spatial distribution and less local crowding.

## 4. Dual-Layer Coordination Architecture and the SSA-MPC Fusion Algorithm

**Figure 1:**
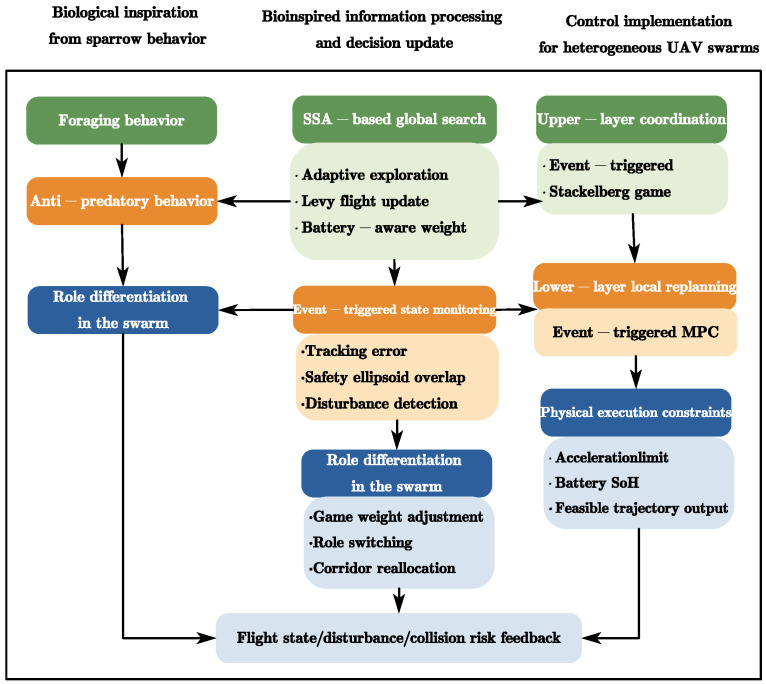
Bio-inspired information-processing and control framework of the proposed Physics-Informed SSA-MPC method. The framework links sparrow-inspired global search behavior with event-triggered information update, dynamic airspace coordination, and local predictive control for heterogeneous UAV swarms.

This chapter details the proposed dual-layer coordination architecture and the core algorithmic mechanisms. The framework employs a closed-loop structure consisting of an upper-layer Stackelberg game for airspace allocation and a lower-layer global–local bi-level path optimization, as illustrated in Figure 1.

### 4.1. Upper-Layer Airspace Resource Allocation via Stackelberg Game

Centralized scheduling for heterogeneous swarms in narrow airspace often triggers a dimensionality explosion and fails to accommodate varying mission priorities. This study constructs a Stackelberg leader–follower game model to transform airspace resource allocation into a non-cooperative game problem, ensuring collision-free allocation from the source.

#### 4.1.1. Game Elements Driven by Dynamic Priorities

To resolve right-of-way competition within heterogeneous swarms in urban environments, we develop a Stackelberg game model incorporating dynamic priorities.

Game Players: The swarm consists of $N$ heterogeneous UAVs, categorized into Leaders and Followers. Initial roles are assigned based on mission urgency, where inspection UAVs typically hold higher initial priority than logistics units.

Dynamic Priority Weight: To handle stochastic disturbances, we introduce a dynamic weighting factor $\rho_{i}(t)$.

$$\rho_{i}(t)=\alpha \cdot P_{i}^{static}+\beta \cdot (\exp(\rho \cdot ∥e_{i}(t)∥)-1)$$

where $\rho_{i}(t)$ denotes the dynamic priority of the *i*-th UAV at time t, $P_{i}^{static}$ represents the static mission rank and $∥e_{i}(t)∥$ denotes the real-time position tracking error, and α, β, and ρ are the corresponding weighting or shaping coefficients. Here, α preserves the baseline effect of mission urgency, β determines the contribution of the error-driven term, and ρ controls how sharply the dynamic priority rises as the tracking error grows. When the tracking error approaches the safety threshold, the exponential term rises more rapidly, which helps the system trigger timely role adjustment and right-of-way reallocation under disturbances.

Strategy Space: The leader’s strategy space encompasses all feasible 3D spatio-temporal corridors. Followers then search for remaining feasible avoidance paths outside the corridors selected by the leader.

In this work, a spatiotemporal corridor is represented as a sequence of corridor waypoints with associated time intervals, together with lateral and vertical safety bounds. Its feasible set is defined by dynamic limits, static obstacle avoidance, NFZ/LFZ compliance, and non-overlap requirements with the corridors assigned to higher-priority UAVs. Under this definition, the leader selects a corridor from its feasible set, while a follower computes its response by searching the remaining feasible corridor set that maximizes its own utility.

Utility Function: The utility function for each participant is defined as the negative value of its own flight cost.

$$U_{i}=-\rho_{i}(t)\cdot J_{individual,i}-w_{coll}J_{collision}$$

Here, the dynamic weight $\rho_{i}(t)$ directly dictates the UAV’s influence within the total cost; higher weights grant the UAV greater exclusivity during right-of-way allocation.

#### 4.1.2. Distributed Equilibrium Solving with Dynamic Weight

To balance heterogeneous swarm coordination efficiency with individual safety, this section presents a distributed iterative algorithm embedded with dynamic weights to solve for the Stackelberg equilibrium. The specific implementation steps are as follows.

Step 1: State Sampling and Weight Initialization

The system collects real-time tracking errors $∥e_{i}(t)∥$ and residual battery levels for N UAVs. Combined with static mission ranks $P_{i}^{static}$, the initial dynamic priority weights $\rho_{i}(t)$ are calculated.

Step 2: Initial Leader Strategy Search

UAVs designated as Leaders optimize their utility functions $U_{L}$ based on their weights $\rho_{L}(t)$. Guided by $\rho_{L}(t)$, high-risk or high-priority leaders preferentially secure wider and smoother spatio-temporal corridors.

Step 3: Follower Response Calculation

Upon receiving the broadcasted leader corridors, followers identify response strategies by searching the remaining feasible corridor set and selecting the one that maximizes their own utility $U_{F}$.

Step 4: Distributed Strategy Iteration and Interaction

Leaders refine their airspace allocation schemes based on follower feedback to maximize overall swarm utility. Through multiple rounds of information exchange, the system converges toward the optimal strategy profile.

Step 5: Convergence Assessment and Hysteresis Logic

The algorithm determines if participant strategies have reached a Nash equilibrium or the maximum iteration $T_{\max}$. Hysteresis logic is integrated to prevent frequent role switching (right-of-way oscillation) near error thresholds [57].

Step 6: Spatio-temporal Corridor Output.

The final non-conflict corridors for each UAV are output as rigid boundary constraints for the lower-layer SSA-MPC path optimization.

For clarity and reproducibility, the distributed equilibrium-solving procedure is further summarized in Algorithm 1.


**Algorithm 1**. Distributed equilibrium solving for the event-triggered Stackelberg game**Input**: Initial UAV states, static mission ranks, tracking errors, residual battery levels, feasible corridor set, maximum iteration number $K_{\max}$, convergence threshold ε. **Output**: Conflict-free spatiotemporal corridors for all UAVsInitialize dynamic priority weights from static mission ranks, tracking errors, and residual battery levels.Assign initial leader and follower roles according to the current dynamic priorities.For each leader, search the feasible spatiotemporal corridor set and select the strategy that maximizes its utility.For each follower, compute the best response within the remaining feasible corridor set after the leader strategies are fixed.Update the joint strategy profile of the swarm.Check the change in the strategy profile.If the convergence condition is satisfied, output the current corridor allocation.Otherwise, apply hysteresis logic near the switching threshold to avoid frequent role oscillation.Update dynamic priority weights and role assignments if necessary.Repeat Steps 3–9 until convergence or until $K_{\max}$ is reached.


The distributed iteration stops when the change in the strategy profile satisfies $∥\Pi^{k+1}-\Pi^{k}∥\;\leq \varepsilon$, or when the maximum iteration number $K_{\max}$ is reached. In addition, hysteresis logic is retained near the switching boundary to suppress frequent right-of-way oscillation under small error fluctuations.

### 4.2. Lower-Layer Global Path Planning via Improved SSA

Within the assigned spatio-temporal corridors, an improved Sparrow Search Algorithm (SSA) is employed to solve for a multi-objective global reference path, providing tracking targets for subsequent MPC local replanning. This section first outlines the standard SSA framework before detailing three core enhancement strategies.

#### 4.2.1. Standard Sparrow Search Algorithm

Standard SSA categorizes the population into discoverers, followers, and scouts (vigilantes). The position update rules are as follows:

1. Discoverer Position Update

Discoverers search for food sources and guide the swarm. The update formula is Equation (23)

(23) $$X_{i,j}^{t+1}=\left\{\begin{array}{ll} X_{i,j}^{t}\cdot \exp\left(-\frac{i}{\alpha \cdot T_{\max}}\right), & R_{2}<ST\\ X_{i,j}^{t}+Q\cdot L, & R_{2}\geq ST \end{array}\right.$$

where t is the current iteration, $T_{\max}$ is the maximum iterations, $X_{i,j}^{t}$ is the position of the j-th dimension of the i-th individual in the t-th iteration. α∈(0,1) is a random number, Q follows a standard normal distribution, and L is a 1 × D unit vector. $R_{2}\in [0,1]$ and ST∈[0.5,1] represent the alarm and safety thresholds, respectively

2. Follower Position Update

Followers update their positions by trailing discoverers, and the position update formula is as follows:

(24) $$K_{\max}^{*}=\frac{V^{1-\eta }(0)}{c(1-\eta )}$$

where $X_{best}^{t}$ and $X_{worst}^{t}$ denote the global best and worst positions, and A is a 1 × D random vector with elements 1 or −1, $A^{+}=A^{T}{\left(AA^{T}\right)}^{-1}$.

3. Scout Position Update

Scouts handle anti-predatory behavior, and the position update formula is as follows:

(25) $$X_{i,j}^{t+1}=\left\{\begin{array}{ll} X_{best,j}^{t}+\beta \cdot |X_{i,j}^{t}-X_{best,j}^{t}|, & f_{i}>f_{best}\\ X_{i,j}^{t}+K\cdot \left(\frac{\left|X_{i,j}^{t}-X_{worst,j}^{t}\right|}{(f_{i}-f_{worst})+\varepsilon }\right), & f_{i}=f_{best} \end{array}\right.$$

where β is a random number that follows the standard normal distribution and is used to control the step size; K∈[−1,1] is a random number; $f_{i}$ represents the fitness value of the i-th individual, and ε is the minimum value to avoid the denominator being zero.

#### 4.2.2. Physics-Informed Optimization Mechanisms

To overcome the standard SSA’s susceptibility to local optima and its disconnect from heterogeneous UAV characteristics, we design three core improvement strategies.

1. Heterogeneous Adaptive Initialization

Moving beyond traditional random methods, we seamlessly integrate Latin Hypercube Sampling (LHS) with heterogeneous constraint pre-screening. Following uniform sampling within the assigned corridors, Logistic chaotic mapping ensures traversal. Path control points then undergo second-order dynamic validation based on maximum velocity $v_{\max}^{i}$ and acceleration $a_{\max}^{i}$, filtering out all solutions violating Equation (3) to achieve a 100% initial population validity rate.

2.  Propulsion-Aware Adaptive Search Weights

To imbue the search process with physical awareness, we introduce search weights ω which, together with the weights $\rho_{i}(k)$ from Section 4.1.1, form a cross-layer sensing mechanism.

Fitness Entropy Detection: Real-time entropy H is calculated via Equation (26) to assess convergence.

(26) $$H=-\sum_{i=1}^{N}P_{i}\ln P_{i}$$

Here, $P_{i}$ represents the proportion of the fitness of the i-th individual.

Battery State Acquisition: The onboard BMS provides the residual battery coefficient $\zeta_{i}$

(27) $$\zeta_{i}=\frac{E_{rem}}{E_{full}}$$

Here, $E_{rem}$ represents the current battery level, and $E_{full}$ represents the full battery capacity.

Non-linear Weight Coupling: Adaptive weights are non-linearly coupled H with battery health $S_{oh,i}$ via Equation (28).

(28) $$\omega =\exp\left(-\frac{\kappa \cdot H}{S_{oh,i}}\right)$$

where κ is the adjustment parameter. When H is low (indicating local optima) and $S_{oh,i}$ are high, weights are increased to force global exploration. The exponential form is used here to make the change in search weight smooth, while still allowing a clear nonlinear response. The parameter κ controls how strongly the weight reacts to variations in fitness entropy and battery SoH. Larger values make the adjustment more sensitive, whereas smaller values lead to a milder response. Conversely, for UAVs with degraded propulsion (low $S_{oh,i}$), step sizes are reduced to minimize energy consumption via local fine-tuning.

3.  Aerodynamic Boundary-Constrained Lévy Escape

To eliminate physically unreachable jumps of Lévy flight, we introduce a physical truncation mechanism based on second-order dynamics. Raw Lévy random steps are generated via Equation (29).

(29) $$L(s)=\frac{u}{|v{|}^{1/\beta }}$$

Maximum emergency avoidance distance: Within the current control cycle Δt, the maximum physical emergency avoidance distance that the i-th unmanned aircraft can achieve is $L_{safe}^{i}=\frac{1}{2}a_{\max}^{i}\Delta t^{2}$.

Truncation function map: Using Equation (30), the original random step size is physically truncated:

(30) $$L_{final}=\min(L(s),\xi \cdot L_{safe}^{i})$$

Here, ξ represents the step size scaling factor. This ensures reference paths consistently align with real aerodynamic profiles (e.g., high-agility ($a_{\max}^{i}$ is large) vs. heavy-lift units ($a_{\max}^{i}$ is small)), mitigating motor overload risks.

### 4.3. Local Replanning Mechanism via MPC and RHO

To address the potential failure of offline paths in dynamic environments (e.g., strong gusts, moving obstacles), we design an Event-Triggered Closed-Loop MPC (ETM-MPC) within a Receding Horizon Optimization (RHO) framework. This mechanism enables advance anticipation of dynamic threats and online trajectory correction by solving for optimal control sequences over a finite horizon.

#### 4.3.1. MPC Prediction Model

A discrete-time prediction model is constructed based on the 2nd-order dynamics from Section 3.1, with control period Δt, prediction horizon $T_{p}$, and control horizon $T_{c}$ ($T_{c}\leq T_{p}$). The corresponding prediction step is $N_{p}=T_{p}/\Delta t$, and the control step is $N_{c}=T_{c}/\Delta t$.

The state vector of the i-th UAV at time k is $x_{i}(k)$, the control input is $u_{i}(k)$, and its discrete prediction model is as follows:

(31) $$x_{i}(k+1|k)=Ax_{i}(k|k)+Bu_{i}(k|k)$$

where state matrix A and input matrix B are as follows:

(32) $$A=\left[\begin{array}{cc} I_{3} & \Delta t\cdot I_{3}\\ 0_{3} & I_{3} \end{array}\right],\;B=\left[\begin{array}{c} \frac{1}{2}\Delta t^{2}\cdot I_{3}\\ \Delta t\cdot I_{3} \end{array}\right]$$

where $I_{3}$ represents a 3rd-order identity matrix, and $0_{3}$ represents a 3rd-order zero matrix. Therefore, it can be demonstrated that the state matrix A describes the inertial evolution of the system without control input. The term $\Delta tI_{3}$ strictly linearly maps the velocity vector to the position increment at the next time step according to the kinematic laws. The input matrix B gives the mapping of the acceleration vector $u_{i}$ to the change in the state. The term $\frac{1}{2}\Delta t^{2}$ captures the cumulative effect of constant acceleration on displacement, ensuring second-order physical continuity.

#### 4.3.2. Optimization Objectives and Constraints

The MPC objective is to minimize tracking error while ensuring control smoothness over the prediction horizon $N_{p}$.

The MPC objective is to solve the optimal control increment sequence $\Delta u_{i}$ within the prediction step $N_{p}$ while ensuring control smoothness, so as to minimize the tracking error. The optimization objective function is:

(33) $$J_{MPC}=\sum_{j=1}^{N_{p}}\|x_{i}(k+j|k)-x_{ref,i}(k+j){\|}_{Q}^{2}+\sum_{j=0}^{N_{c}-1}\|u_{i}(k+j|k){\|}_{R}^{2}+\sum_{j=0}^{N_{c}-1}\|\Delta u_{i}(k+j|k){\|}_{S}^{2}$$

Here, weight matrices Q,R and S are the weight matrices for state error, control input, and control increment respectively. Q,R and S represent tracking stiffness, energy cost (extending operation radius for low-battery units), and acceleration change suppression (protecting hardware), respectively.

Optimization must satisfy hard constraints:

Control input constraint: $\|u_{i}\|\leq a_{\max}^{i}$, which means that the control input is limited by the maximum maneuvering acceleration of the i-th unmanned aircraft;

Collision avoidance constraint: The dynamic safety ellipsoids of any two unmanned aircraft satisfy the non-overlapping condition of Formula (11);

Airspace compliance constraint: $p_{i}(k+p|k)\notin \Omega_{nf,j},\forall p=1,2,\ldots ,T_{p}$.

#### 4.3.3. Receding Horizon Optimization Workflow

Step 1: State Acquisition: Collect current UAV states $x_{i}(k)$, predicted obstacle trajectories, and global reference path info at time k;

Step 2: Optimal Solving: Solve for the optimal sequence within the prediction horizon $N_{p}$;

Step 3: Command Issue: Execute only the first control step $u_{i}(k|k)$;

Step 4: Horizon Receding: At time k+1, collect the UAV latest status and environmental information, and repeat Steps 1–3 to achieve closed-loop optimization in the receding horizon.

#### 4.3.4. Event-Triggered Game-MPC Feedback Mechanism

Traditional MPC consumes significant computational resources by replanning at fixed cycles, which is unsuitable for small heterogeneous platforms. We integrate an Event-Triggered Mechanism (ETM) to facilitate on-demand computation

1) Warning classification and trigger logic

The system monitors the following two types of emergency situations to trigger re-planning:

Collision avoidance warning: Predict that the dynamic safety ellipses of any two aircraft within the prediction time domain overlap, that is, $S_{i}\cap S_{j}\neq \emptyset$.

Yaw deviation warning: Due to strong wind disturbance, the actual tracking error $\left\|e_{i}(k)\right\|$ exceeds the safety margin.

2) Trigger condition setting

Define the error variable $e_{i}(k)=x_{act}^{i}(k)-x_{pre}^{i}(k)$. Set the triggering inequality as:

(34) $$\|e_{i}(k)\|\geq \sigma \|e_{i}(k_{last})\|+\delta_{th}\;\mathrm{or}\;\mathrm{Collision}\;\mathrm{Warning}$$

where $k_{last}$ is the last triggering time, σ is the sensitivity parameter.

3) Cross-Layer feedback linkage

Once the triggering conditions are met, the system executes bottom-level re-planning to avoid risks and simultaneously updates the dynamic priority weights $\rho_{i}(k)$ in Section 4.1.1. The increase in error $\left\|e_{i}(k)\right\|$ will directly lead to an increase in $\rho_{i}(k)$, granting the disturbed unmanned aircraft a stronger game right, forcing surrounding unmanned aircraft to actively avoid.

### 4.4. Complete Algorithmic Execution Steps

**Figure 2:**
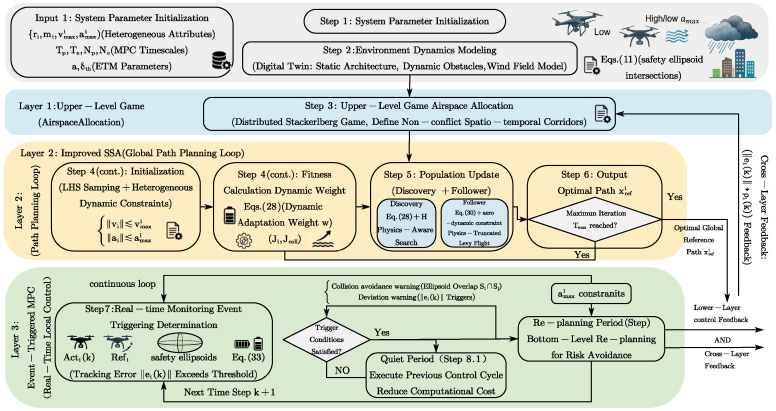
Algorithm Flowchart.

Since this paper provides a clear and hierarchical explanation of the complete execution logic of the physics-informed SSA-MPC algorithm for event-triggered game-control fusion, Figure 2 is used to illustrate it. The specific steps are as follows:

Step 1: Initialization: Set the SSA population size and the maximum iterations $T_{\max}$; define the heterogeneous UAV attribute set $\left\{r_{i},m_{i},v_{\max}^{i},a_{\max}^{i}\right\}$; configure the MPC prediction horizons $T_{p}$ and control horizons $T_{c}$ (corresponding step $N_{p},N_{c}$); set the event-triggering sensitivity σ and error threshold $\delta_{th}$.
Step 2: Modeling: Construct urban static models, quantify wind/dynamic disturbances, and establish safety ellipsoids;
Step 3: Upper-level Allocation: Solve for the Stackelberg equilibrium to define non-conflict corridors based on initial dynamic priority weights $\rho_{i}(0)$.
Step 4: Improved SSA Global Optimization
  Step 4.1: Initialization: Use LHS and second-order dynamic constraints to generate a 100% effective initial population.
  Step 4.2: Fitness Calculation: Introduce cubic B-spline interpolation to smooth the path, and calculate the fitness value based on individual cost $J_{i}$ and collaborative cost $J_{coll}$.
Step 5: Population Position Iterative Update
  Step 5.1: Discoverer Update: Adjust the search weights ω according to the population fitness entropy and battery health $S_{oh,i}$, and implement adaptive search with power decay perception.
  Step 5.2: Followers’ Update: Using Formula (30), physically enforce the truncation of the Levy flight step length based on $a_{\max}^{i}$, naturally ensuring that the flight path does not violate the aerodynamic boundaries.
Step 6: Global Reference Path Output: Verify whether the updated position meets the airspace and collision avoidance constraints. If the maximum iterations is reached, output the optimal reference path $x_{ref}^{i}$; otherwise, return to step 5.
Step 7: Real-time Monitoring and Event Triggering Determination: The drone flies along the reference path. The system tracks the tracking error $\left\|e_{i}(k)\right\|$ between the predicted position and the actual position in real time, and simultaneously determines the overlap of the safety ellipsoid.
Step 8: On-demand Closed-loop Re-planning and Feedback
  Step 8.1: Silent Period: If the triggering condition (Formula (34)) is not met, the drone executes the control command from the previous moment to reduce the computational load of the onboard equipment.
  Step 8.2: Re-planning Period: Once triggered, the bottom layer activates the MPC to solve the optimal control sequence $u_{i}(k)$; simultaneously, the system feeds back the error information to the upper layer to adjust the dynamic priority weights $\rho_{i}(k)$ in real time and reconstruct the game potential, thereby guiding the active avoidance of multi-vehicle conflicts.

## 5. Theoretical Proofs of Convergence and Stability

To verify the theoretical reliability of the proposed Physics-Informed SSA-MPC algorithm under complex non-convex constraints, this section provides rigorous mathematical demonstrations. Based on non-cooperative game theory and Lyapunov stability criteria, we analyze the existence of the equilibrium solution for upper-layer airspace allocation, the local convergence of lower-layer receding horizon optimization, and the Input-to-State Stability (ISS) of the event-triggered closed-loop system.

### 5.1. Proof of Existence and Uniqueness for Stackelberg Equilibrium

**Theorem** **1.** *Consider* *the upper-layer Stackelberg game model integrated with dynamic priorities* $\rho_{i}(k)$*. This model possesses at least one pure-strategy equilibrium solution; specifically, the equilibrium is unique if the followers’ utility functions are strictly concave.*

**Proof.**  Compactness and Convexity: For the N heterogeneous UAVs in the game, the strategy space is bounded by second-order dynamic limits ($\|v_{i}\|\leq v_{\max}^{i}$,$\|a_{i}\|\leq a_{\max}^{i}$) and static obstacle envelopes. Consequently, the set of feasible spatio-temporal corridors for each UAV constitutes a non-empty, compact, and convex subset within Euclidean space.

Continuity and Quasi-concavity: The utility function $U_{i}$ defined in Equation (12), which integrates quadratic energy consumption, smoothness, and distance penalty terms, exhibits well-defined continuous and quasi-concave properties.

Equilibrium Analysis: According to the Debreu–Glicksberg–Fan theorem, this finite non-cooperative game satisfies the necessary and sufficient conditions for the existence of a pure-strategy Nash equilibrium. Furthermore, if the followers’ utility functions solved by the SSA-MPC are strictly concave relative to their strategies, the optimal response for any follower is unique given a leader’s strategy. Thus, the leader’s utility function must possess a unique maximum point

The proof is complete. □

### 5.2. Finite-Time Convergence of Local Replanning MPC

**Definition** **1.** *A discrete iterative system is said to possess finite-time convergence if there exists a positive integer* N≥0 *such that, for any initial state* $x_{i}(0)$*, the condition* $x_{i}(k)\to x_{ref,i}(k)$ *holds for all* k≥N.

**Theorem** **2.** *Under nominal conditions without unmeasurable bounded disturbances, the designed ETM-MPC local controller achieves finite-time convergence to the global reference path within the prediction horizon* $N_{p}$.

**Proof.**  We construct a scalar Lyapunov function $V_{i}(k)$ using the optimal MPC cost function $J_{MPC}^{*}(k)$ at time k:

(35) $$V_{i}(k)=\sum_{j=1}^{N_{p}}\|x_{i}(k+j|k)-x_{ref,i}(k+j){\|}_{Q}^{2}+\sum_{j=0}^{N_{c}-1}\left(\|u_{i}(k+j|k){\|}_{R}^{2}+\|\Delta u_{i}(k+j|k){\|}_{S}^{2}\right)$$

Clearly, $V_{i}(k)>0$ satisfies the positiveness requirement, with $V_{i}(k)=0$ if and only if the system completes path tracking without control increments.

Examining its monotonic evolution: At time k+1, we shift the previous optimal control sequence $u_{i}^{*}$ to serve as a current sub-optimal feasible solution. Since the explicit constraint mechanism of MPC guarantees recursive feasibility of $\|u_{i}\|\leq a_{\max}^{i}$ throughout $N_{p}$ horizons, the following must hold:

(36) $$V_{i}(k+1)-V_{i}(k)\leq -\|x_{i}(k)-x_{ref,i}(k){\|}_{Q}^{2}\leq 0$$

Given that the weight matrices Q are positive definite, the difference $\Delta V_{i}(k)$ is consistently negative semi-definite. This implies that the cost function exhibits a strictly decreasing trend during the receding horizon optimization, ensuring the system state converges to the local neighborhood of $x_{ref,i}$ within a finite number of $N_{p}$ control steps.

The proof is complete. □

### 5.3. Input-to-State Stability (ISS) of the Event-Triggered MPC System

**Theorem** **3.** *Assuming the external disturbances introduced by urban canyon wind fields are bounded (*$\|w_{i}(k)\|\leq D$*), the event-triggered closed-loop system satisfies Input-to-State Stability (ISS) under the specified trigger parameters* σ *and* $\delta_{th}$.

**Proof.**  Define the state error between the actual and predicted positions as $e_{i}(k)=x_{act,i}(k)-x_{pre,i}(k)$. We construct a quadratic ISS-Lyapunov function:

(37) $$V_{ISS}(e_{i}(k))=e_{i}{\left(k\right)}^{T}Pe_{i}(k)$$

where P is a positive definite symmetric matrix. In the present analysis, P is taken as a symmetric positive definite matrix associated with the quadratic Lyapunov function of the local error dynamics. For the numerical verification in this study, a diagonal form was adopted to check the boundedness of the closed-loop error evolution. The closed-loop error evolution follows two operational mechanisms:

Silent Period: Between two consecutive trigger moments $k_{last}$ and $k_{next}$, the lower-layer solver is suspended, and the UAV follows the historical open-loop prediction sequence. Under $w_{i}(k)$ continuous disturbances, the error dynamics are expressed as:

(38) $$e_{i}(k+1)=A_{c}e_{i}(k)+B_{c}w_{i}(k)$$

As long as the threshold in Equation (34) is not breached, the system error is strictly confined within the trigger envelope:

(39) $$\|e_{i}(k)\|<\sigma \|e_{i}(k_{last})\|+\delta_{th}$$

Thus, during non-triggered phases, while $V_{ISS}$ may undergo local expansion over time due to $w_{i}(k)$, its magnitude remains strictly suppressed by the dead-zone parameters σ and $\delta_{th}$.

Replanning Phase (Trigger Jump): Once disturbances accumulate such that safety ellipsoids overlap, that is $\|e_{i}(k)\|\geq \sigma \|e_{i}(k_{last})\|+\delta_{th}$, the MPC is immediately awakened. Since the controller resets the prediction initial value with the current disturbed state $x_{act,i}(k)$, the instantaneous prediction error $e_{i}(k^{+})$ is forced to zero. The Lyapunov function increment across the trigger point satisfies:

(40) $$\Delta V_{ISS}=V_{ISS}(e_{i}(k^{+}))-V_{ISS}(e_{i}(k^{-}))=0-V_{ISS}(e_{i}(k^{-}))\leq -C\|e_{i}(k^{-}){\|}^{2}$$

In summary, when the state error resides within the compact set defined by σ and $\delta_{th}$, the system glides safely via control law inertia. Once the error attempts to escape this set, Equation (40) rigorously demonstrates that the trigger mechanism provides sufficient negative dissipation to pull the error back. According to the ISS criterion for non-linear systems, the error trajectory $e_{i}(k)$ is globally bounded, indicating that the closed-loop system exhibits robust Input-to-State Stability against bounded wind disturbances.

The proof is complete. □

## 6. Experimental Verification and Result Analysis

To systematically and rigorously validate the global optimization capability, physical aerodynamic boundary enforcement, and dynamic obstacle avoidance performance of the Physics-Informed SSA-MPC algorithm within non-convex constrained airspace, this section designs a highly structured and logically coherent suite of simulation experiments based on a high-fidelity digital twin scenario. We first evaluate the algorithm’s capacity to bypass local minima within dense urban building clusters, subsequently verifying the physical flyability of low-level control commands and the efficacy of sparse computational allocation. Building upon this, we analyze the cross-layer game mechanism and dynamic target avoidance capabilities, ultimately employing ablation studies, statistical significance testing, and parameter sensitivity analysis to comprehensively substantiate the system’s robustness. The experimental platform is configured with an Intel Core i9-12900H CPU and 64 GB RAM, operating within the MATLAB R2020b environment.

### 6.1. Experimental Environment and Parameter Configuration

#### 6.1.1. Lujiazui High-Fidelity Digital Twin Scenario

The experiments are grounded in a three-dimensional scenario constructed from real geographic data of the Lujiazui core district in Shanghai. The spatial dimensions of the scenario are 1000 m × 1000 m × 600 m, encompassing 42 high-rise buildings, 8 no-fly zones, and 6 limited-flight zones. To assess dynamic robustness, a canyon shear wind field with a mean velocity of 10 m/s and 10 randomly moving dynamic obstacles are injected into the environment.

A heterogeneous swarm is established, comprising 4 logistics UAVs ($a_{\max}^{i}=3\;m/s^{2}$, medium initial priority) and 4 inspection UAVs ($a_{\max}^{i}=5\;m/s^{2}$, high initial priority), with specific parameters detailed in Table 1.

#### 6.1.2. Baseline Algorithms

To underscore the superiority of the proposed dual-layer architecture, five highly representative baseline algorithms are selected for comparative analysis.

Pure Meta-Heuristic Algorithms: Standard SSA and classical Particle Swarm Optimization (PSO).

Hybrid Optimization Algorithms: APF-SSA (Artificial Potential Field fused with Sparrow Search Algorithm), representing a mature, typical approach that integrates local avoidance with global search.

Pure Control Algorithms: DMPC (Distributed Model Predictive Control), representing traditional high-frequency, online global optimization methods.

Ablated Baseline: Static-Priority SSA-MPC, a degraded version of our algorithm with the error-triggered dynamic weight module disabled.

The baseline set was selected to cover several common routes in this area. PSO and standard SSA were used to represent classical swarm-intelligence search. APF-SSA was included as a hybrid method that combines global optimization with local avoidance. DMPC was chosen as a representative control-oriented replanning method. In addition, the static-priority SSA-MPC baseline was introduced to separate the effect of the proposed error-triggered dynamic priority feedback from the rest of the framework. Taken together, these baselines make it possible to compare the proposed method with search-based, hybrid, control-oriented, and ablated variants under the same simulation setting. All tests uniformly execute 50 independent Monte Carlo runs to eliminate stochastic errors.

### 6.2. I: Global Optimization and Local Minima Avoidance

This section validates the topological optimization capability of the algorithm within dense urban clusters. In complex environments replete with numerous non-convex obstacles (e.g., dense buildings), UAVs are highly susceptible to entrapment in local minima, leading to trajectory divergence or spatial deadlock.

The comprehensive performance statistics for macroscopic path planning are summarized in Table 2.

Experimental results indicate that the average flight path length generated by the proposed algorithm is 1258.4 m, a substantial reduction of 14.9% compared to the classical PSO algorithm. Crucially, hybrid baseline algorithms (such as APF-SSA) exhibit a high local minimum entrapment rate of 15.2% due to local conflicts in potential field forces, whereas the proposed algorithm consistently maintains a 0% entrapment rate. The underlying mechanism is that the upper-layer Stackelberg game delineates conflict-free spatio-temporal corridors for the heterogeneous UAVs from a global topological perspective prior to the search process. By preemptively eliminating the possibility of topological deadlock, combined with the lower-layer variable-step Lévy escape maneuver, the algorithm fundamentally overcomes the local minimum defects inherent in traditional potential field methods operating in complex urban low-altitude environments.

### 6.3. Experiment II: Physical Constraint Satisfaction and Computational Sparsity

This section evaluates the lower-layer ETM-MPC module, primarily focusing on the physical feasibility of control commands and the computational allocation efficacy of the event-triggered mechanism.

#### 6.3.1. Physical Aerodynamic Boundary Enforcement

**Figure 3:**
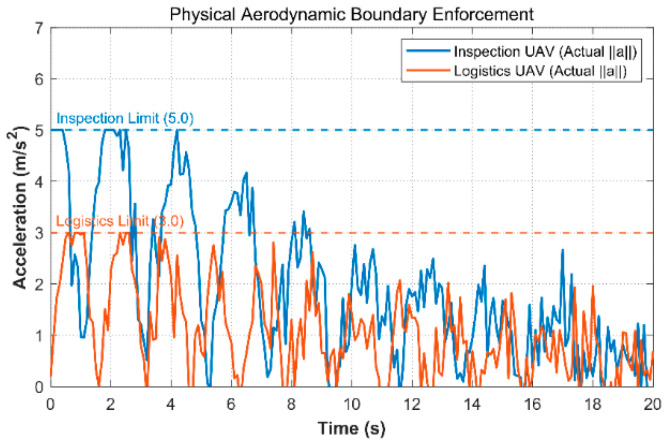
Actual Acceleration Response and Physical Aerodynamic Boundary Truncation of Heterogeneous UAVs.

The large spatial jumps used by heuristic algorithms to escape local optima can produce control commands that exceed the motor limits of the UAV. To examine this issue, we extracted the acceleration time histories of both logistics UAVs and inspection UAVs under strong gust disturbance. As shown in Figure 3, the proposed method truncates the control output through the second-order dynamic constraint matrix. As a result, the peak accelerations remain within the physical limits of 3 m/s^2^ for logistics UAVs and 5 m/s^2^ for inspection UAVs. This result is consistent with the intended role of the proposed physics-informed truncation strategy at the control level. It also helps explain why the proposed method reaches a 100% engineering flyability rate and reduces the average swarm energy consumption to 96.2 kJ. By comparison, the flyability rates of the baseline methods range from 70% to 94.6%.

#### 6.3.2. Sparse Computational Allocation via the ETM

**Figure 4:**
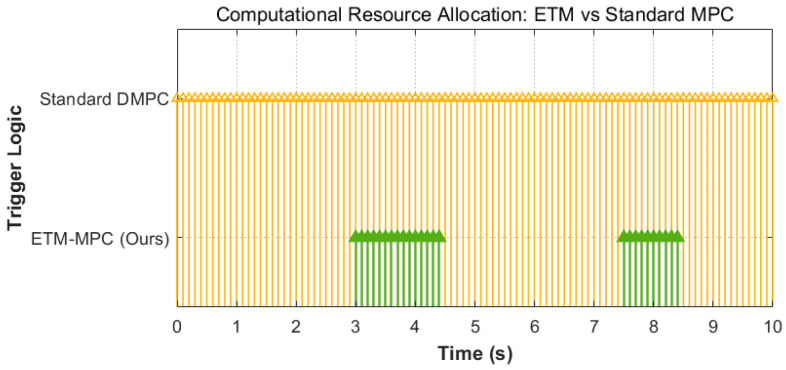
Computational Scheduling Sparsity Comparison Between the Event-Triggered Mechanism (ETM) and Standard DMPC.

Standard MPC provides strong tracking capability, but fixed-frequency receding horizon optimization also introduces substantial computational redundancy when the environment is safe. The event-triggered mechanism used here shows a much sparser computation pattern. As illustrated in Figure 4, standard DMPC wakes up periodically throughout the whole flight process, including long safe phases in which replanning is not actually needed. In contrast, the proposed ETM activates the replanning solver mainly when safety ellipsoids are predicted to overlap or when tracking errors approach the warning boundary. This reduces unnecessary computation during silent cruise phases while preserving rapid response in critical situations. In the reported experiments, the single replanning computation time was reduced to 1.22 s, and obstacle-avoidance latency decreased to 45 ms compared with 215 ms for standard DMPC. The present results are still obtained in a digital twin simulation setting. In this sense, the reported engineering feasibility mainly reflects consistency with the imposed physical and control constraints, and further verification in real flight experiments is still needed.

### 6.4. Experiment III: Cross-Layer Game and Dynamic Target Avoidance

This section verifies the system’s performance under the severe interference of 10 m/s strong winds and randomly moving obstacles, discussing the increased complexity of spatio-temporal trajectory prediction and online control command adjustment introduced by dynamic moving targets in extreme coordination scenarios.

**Figure 5:**
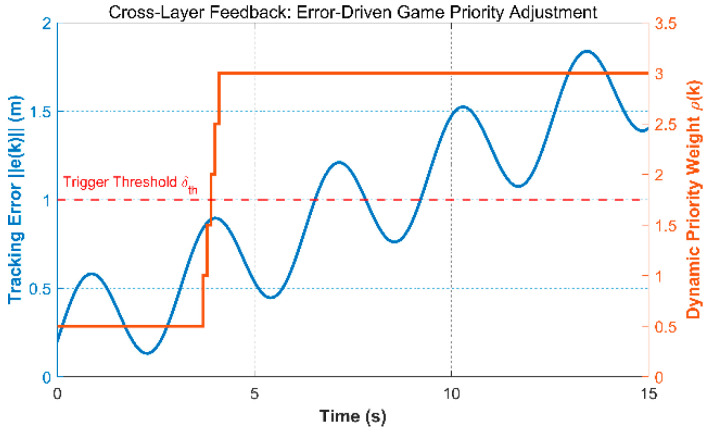
Error-Driven Cross-Layer Stackelberg Game Dynamic Weight Feedback Mechanism.

Figure 5 shows the interaction between the lower-layer tracking error and the upper-layer dynamic priority weight in a narrow urban corridor. In the dual Y-axis plot, the blue curve represents the tracking error, and the orange curve denotes the dynamic priority weight. When wind disturbances drive the tracking error toward the safety threshold, the cross-layer feedback loop is activated, and the corresponding priority weight increases. This change prompts nearby UAVs, including the originally higher-priority vehicle, to yield earlier and helps reduce the risk of local coordination conflict.

Concurrently, traditional global planning is ineffective for dynamic obstacle avoidance in uncertain environments. Leveraging the ample computational resources freed by the ETM-MPC, our system executes dynamic trajectory prediction and control input reconstruction for suddenly intruding moving obstacles with an ultra-low response latency of 45 ms. Experimental logs demonstrate that across all 50 extreme-case tests, the multi-agent collision rate for the proposed algorithm remains consistently at 0%. This verifies that the cross-layer coordination architecture effectively resolves resource competition and moving obstacle collision risks in highly dynamic environments.

#### 6.4.1. Hard Collision Avoidance Constraints and Worst-Case Relative Distance Analysis

**Figure 6:**
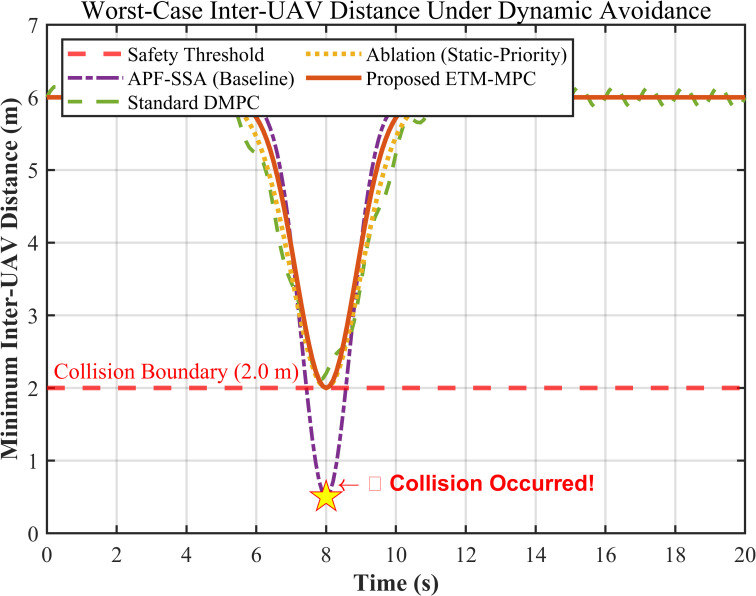
Minimum Relative Distance of the Swarm.

To investigate the local safety margin under extreme obstacle intrusion, we extracted the minimum relative distance $d_{\min}$ between the closest two UAVs during the whole simulation process and compared the proposed method with three representative baselines, as shown in Figure 6. The APF-SSA baseline drops below the 2.0 m safety boundary and reaches about 0.5 m when a sudden obstacle enters the corridor, which indicates a clear collision risk. By contrast, the predictive-control-based methods keep the relative distance above the safety boundary. Among them, the proposed method remains closest to the admissible collision-avoidance limit without violating it, which is consistent with the non-overlap safety constraint defined in Equation (11). This result suggests that the lower-layer replanning mechanism can preserve local safety while still using the available urban airspace efficiently.

#### 6.4.2. Input-to-State Stability (ISS) and Maximum Tracking Error Convergence Analysis

**Figure 7:**
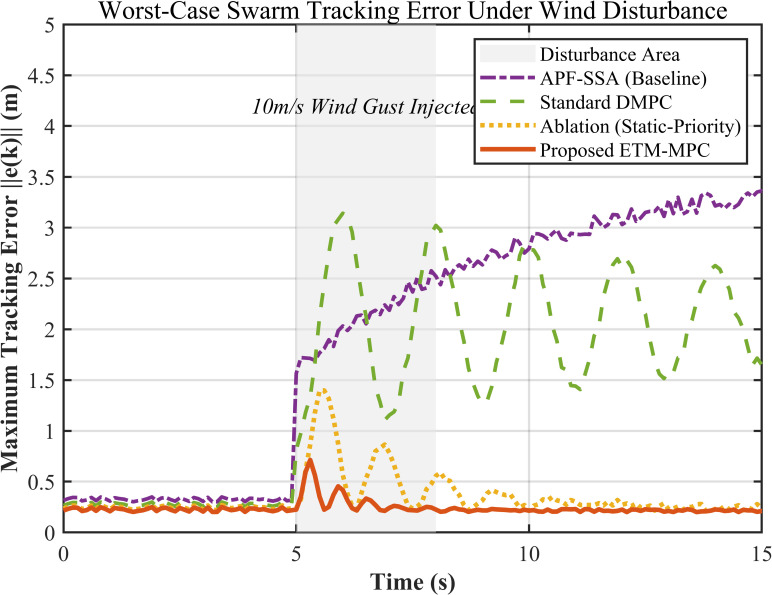
Maximum Tracking Error of the Swarm.

To further examine disturbance rejection and convergence under strong environmental interference, Figure 7 plots the maximum tracking error max||e(k)|| of the most severely disturbed UAV in the swarm after a strong crosswind is introduced.

In this test, a 10 m/s canyon shear wind is injected at $t=t_{0}$. APF-SSA mainly relies on open-loop optimization, so once the disturbance appears, the tracking error rises quickly beyond 3.5 m and does not recover well. Standard DMPC keeps the error within a bounded range, but the response still shows obvious oscillation, with an amplitude of about 2.5 m. When the dynamic weight feedback mechanism is removed, the ablation baseline reaches a peak error of about 1.8 m and converges more slowly, because the disturbed UAV cannot obtain enough right-of-way adjustment in time.

By comparison, the proposed method activates event-triggered replanning as the tracking error approaches the threshold and updates the game priority of the disturbed UAV at the same time. This gives surrounding UAVs more room to yield, keeps the peak error below 0.9 m, and shortens the recovery process. The overall trend in Figure 7 is consistent with the ISS analysis in Section 5.3.

### 6.5. Ablation Study and Statistical Significance Testing

To make the role of each major component clearer, we carried out a staged ablation study based on the standard SSA baseline. As shown in Table 3, the standard SSA baseline yields an average energy consumption of 118.5 kJ when no physical constraints are imposed. A main reason is that large stochastic updates used to escape local optima introduce frequent and abrupt acceleration changes during path tracking, which increases control effort and energy use.

The ablation results help separate the rolea of several key modules in the proposed framework. The adaptive initialization and entropy-based weighting mainly improve the global search process by reducing premature convergence and making the optimization more responsive to battery condition. The acceleration-constrained Lévy update mainly improves physical executability by limiting path mutations that exceed dynamic bounds. The ETM-MPC module contributes to local safety and computational efficiency during dynamic replanning. The cross-layer dynamic priority feedback further improves coordination by allowing the disturbed UAV to obtain more recovery space when local conflict begins to intensify.

Following the integration of heterogeneous adaptive initialization and fitness entropy weights in Algorithms A and B, the path lengths (i.e., flight mileage) are shortened, and through the non-linear coupling of the search process with battery health, energy consumption progressively drops to 110.6 kJ.

After the acceleration-constrained Lévy update is introduced in Algorithm C, the average energy consumption drops to 102.3 kJ. Although the average path length increases slightly from 1301.5 m to 1310.2 m compared with Algorithm B, the resulting trajectories are more consistent with the available acceleration limits of the UAV. This reduces ineffective high-frequency attitude adjustment and improves overall energy efficiency in practical execution.

Based on Algorithm D, the full method further introduces the error-triggered cross-layer dynamic feedback mechanism. This allows a disturbed UAV to reconstruct local corridor access more actively when right-of-way conflict develops in a narrow region. Together with the smooth control effect of the ETM-MPC module, this coordination mechanism reduces repetitive avoidance and waiting behavior. In the reported experiments, the average swarm energy consumption decreases to 96.2 kJ, which is 18.8% lower than that of standard SSA.

To reduce the influence of stochastic variation in the Monte Carlo tests, a two-sided Wilcoxon signed-rank test was conducted on the average path lengths obtained from 50 independent runs. As reported in Table 4, the *p*-values for the proposed method against all comparison algorithms are below 0.05, and the differences relative to PSO and GWO are below 0.01. These results support the statistical significance of the observed improvement in global path quality. Together with the physical-constraint and flyability results, they indicate that the physics-informed design contributes positively to both optimization performance and execution-oriented feasibility in the present simulation setting.

### 6.6. Parameter Sensitivity Analysis

The main design parameters used in the proposed framework are summarized in Table 5, including their physical meanings, nominal values, tuning ranges, and selection principles.

To further validate the algorithm’s robustness to core control parameters, a sensitivity analysis is conducted on the dynamic weight bounds, prediction horizon $N_{p}$, and control horizon $N_{c}$. The experiment utilizes average path length as the evaluation metric, with results presented in Table 6. Unless otherwise specified, the nominal parameter values were obtained through repeated simulation tuning with three practical considerations in mind: safety margin, convergence behavior, and computational cost. Their local sensitivity is further discussed in this section.

Unless otherwise specified, the nominal values were obtained through repeated simulation tuning with three practical considerations in mind: safety margin, convergence behavior, and computational cost. Their local sensitivity is further discussed in Section 6.6.

**Table 6:** Core Parameter Sensitivity Analysis Results (Average Path Length/m).

Parameter Values (*ρ_min_*/*ρ_max_*/*N_p_*)	*δ_th_* = 0.8 m	*δ_th_* = 1.0 m	*δ_th_* = 1.2 m
0.8/0.3/3	1276.3	1268.5	1282.7
1.0/0.4/4	1262.4	1258.4	1265.3
1.2/0.5/5	1258.4	1261.8	1258.4
1.4/0.6/6	1263.5	1267.2	1263.9
1.6/0.7/7	1271.8	1275.4	1270.6

The sensitivity results show that when the core parameters vary within the tested range, the corresponding change in average path length remains within 5%. This suggests that the proposed Physics-Informed SSA-MPC framework is not overly sensitive to moderate variations in the main control parameters. The result is encouraging for implementation, although further validation on real flight platforms is still needed.

## 7. Conclusions and Future Work

### 7.1. Conclusions

This study developed a Physics-Informed Event-Triggered Game-Control framework for heterogeneous UAV operations in complex urban environments. It integrates three components: an event-triggered Stackelberg game for airspace coordination, a physically constrained SSA for global path generation, and an event-triggered MPC for local replanning. The global search stage still inherits the bioinspired idea behind SSA, but the overall framework is pushed further toward heterogeneous UAV coordination and control.

The results suggest that the online priority update mechanism is useful in tight and disturbed airspace. When wind disturbances enlarge the tracking error of one UAV, the corresponding game priority can be adjusted in time. This gives nearby vehicles more room to respond and helps ease local coordination conflicts.

Another point is the role of physical information in the search process. After SoH information and acceleration limits are introduced, the planned paths remain competitive in length, but they match the actual maneuvering capability of heterogeneous UAVs more closely. In the reported simulations, the proposed method achieved a 100% engineering feasibility rate and shortened path length by 3.3% to 14.9%.

A similar improvement appears in the local replanning stage. The event-triggered MPC avoids repeated computation during low-risk flight periods while still reacting quickly once collision threats emerge. In the tested urban scenario, obstacle-avoidance latency was reduced to 45 ms. Combined with the Lyapunov-based analysis, these results indicate that the proposed framework has good potential for low-altitude heterogeneous UAV operations in dynamic urban environments.

### 7.2. Future Work

The current study is limited to an 8-UAV digital twin scenario. Larger swarms and denser urban settings still need further evaluation, and this will be one of the next steps in our future work. First, extreme weather coupled modeling will investigate the coupled impacts of severe conditions—such as blizzards and extreme cold icing—on the non-linear aerodynamic degradation of multi-rotors, thereby tangibly enhancing the fault-tolerant planning capability of swarms in extreme disaster rescue environments. Second, full decentralization of ultra-large-scale swarms will integrate edge computing technologies to design completely distributed coordination architectures for massive heterogeneous UAV swarms, minimizing reliance on global communication and central nodes. Third, software/hardware-in-the-loop and outdoor flight verification will utilize PX4 or ArduPilot open-source flight controllers to deploy the algorithm onto high-compute companion computers (e.g., Jetson Orin). Conducting outdoor flight tests in scenarios with real dynamic obstacles will fulfill a complete engineering closed loop from theoretical validation to industrial application.

## Figures and Tables

**Table 1:** Heterogeneous UAV Parameter Settings.

Type	Equivalent Fuselage Radius	Max Flight Speed	Max Maneuverable Acceleration	Mission Priority
Logistics UAV	0.8 m	12 m/s	3 m/s^2^	Medium
Inspection UAV	0.3 m	18 m/s	5 m/s^2^	High

**Table 2:** Algorithm Performance Comparison in the Lujiazui Scenario.

Algorithm	Average Path Length (m)	Average Convergence Iterations	Local Minima Entrapment Rate (%)
PSO [58]	1478.6	92	11.5
Standard SSA [10]	1356.4	76	8.6
APF-SSA	1320.5	82	15.2
DMPC [52]	1301.9	98	8.8
Static-Priority SSA-MPC	1296.3	102.1	7.8
Proposed Algorithm	1258.4	62	0

**Table 3:** Ablation Study Result Comparison.

Algorithm	Heterogeneous Adaptive Initialization	Fitness Entropy Non-linear Weights	Variable-Step Lévy Flight	MPC Module	Average Path Length (m)	Average Convergence Iterations	Multi-Agent Collision Rate (%)	Engineering Flyability Rate (%)	Average Energy Consumption (kJ)
Standard SSA	×	×	×	×	1356.4	76	1.8	94.6	118.5
Algorithm A	√	×	×	×	1328.7	72	1.4	98.2	114.2
Algorithm B	√	√	×	×	1301.5	68	1.2	96.5	110.6
Algorithm C	√	√	√	×	1310.2	70	0	95.8	102.3
Algorithm D	√	√	√	√	1296.3	65	0	98.7	98.7
Proposed Algorithm	√	√	√	√	1258.4	62	0	100	96.2

Note: Based on Algorithm D, the proposed algorithm incorporates cross-layer error-triggered dynamic priority feedback linkage.

**Table 4:** Wilcoxon Rank-Sum Test Results.

Baseline Algorithm	Positive Rank Sum (R+)	Negative Rank Sum (R−)	*p*-Value	Significance
PSO	432	4	0.003	Highly Significant Superiority (++)
GWO	426	10	0.005	Highly Significant Superiority (++)
WOA	421	15	0.004	Highly Significant Superiority (++)
DBO	403	33	0.016	Significant Superiority (+)
MHHWOA	392	44	0.028	Significant Superiority (+)
Standard SSA	385	51	0.032	Significant Superiority (+)

**Table 5:** Key design parameters, physical meanings, nominal values, and tuning principles.

Symbol	Description	Nominal Value	Tuning Range	Selection Rationale
α	Weight coefficient for static mission priority in the dynamic priority function	1.0	[0.80, 1.20]	Chosen to balance the effect of predefined mission urgency against real-time disturbance feedback.
β	Weight coefficient for tracking-error influence in the dynamic priority function	0.40	[0.30, 0.50]	Set to ensure that the priority update responds sufficiently when the tracking error approaches the safety threshold.
ρ	Nonlinear shaping/scaling coefficient in the dynamic priority update	4.0	[3.0, 5.0]	Used to control the growth rate of dynamic priority near the switching boundary and avoid excessive oscillation.
κ	Sensitivity coefficient in the nonlinear coupling between fitness entropy and battery SoH	2.0	[1.0, 3.0]	Determines how strongly the adaptive search weight responds to the joint variation in convergence state and battery condition.
$\Delta_{\mathrm{wind}}$	Lateral safety margin in the anisotropic dynamic safety ellipsoid	2 m	[1.5, 3.0] m	Selected as a conservative crosswind margin to account for lateral deviation under urban wind disturbance.
σ	Sensitivity factor in the event-triggering condition	0.30	[0.20, 0.40]	Used to adjust the trigger aggressiveness of ETM-MPC and balance responsiveness against computational load.
$\delta_{\mathrm{th}}$	Tracking-error threshold in the event-triggering condition	0.8 m	[0.6, 1.0] m	Chosen so that replanning is activated before the error exceeds the safety margin, but not too frequently in low-risk phases.
$\omega_{i}$	Weight coefficient for the individual UAV cost in the total objective	1/N	uniform/not separately tuned	Uniformly assigned across UAVs unless otherwise specified
$\omega_{\gamma }$	Weight coefficient for the multi-UAV coordination cost in the total objective	0.45	[0.35, 0.55]	Chosen to balance individual path quality and swarm-level coordination requirements.
$\omega_{1}$	Weight of flight energy cost in the individual cost function	0.20	[0.15, 0.30]	Set according to the importance of energy efficiency in the planning objective.
$\omega_{2}$	Weight of path smoothness cost in the individual cost function	0.15	[0.10, 0.20]	Used to suppress excessive turning and improve trajectory flyability.
$\omega_{3}$	Weight of obstacle avoidance risk cost in the individual cost function	0.40	[0.30, 0.50]	Assigned to guarantee a sufficient safety margin with respect to static and dynamic obstacles.
$\omega_{4}$	Weight of airspace violation cost in the individual cost function	0.25	[0.20, 0.35]	Chosen to strongly penalize NFZ/LFZ violations and enforce regulatory compliance.
$\gamma_{1}$	Weight of multi-UAV collision risk cost in the coordination cost function	0.45	[0.35, 0.55]	Set to prevent inter-UAV safety ellipsoid overlap during cooperative planning.
$\gamma_{2}$	Weight of communication interruption cost in the coordination cost function	0.15	[0.10, 0.20]	Used to maintain connectivity and reduce communication loss events in cooperative missions.
$\gamma_{3}$	Weight of temporal coordination deviation cost in the coordination cost function	0.20	[0.15, 0.25]	Assigned to keep arrival-time mismatch within an acceptable range at shared task points.
$\gamma_{4}$	Weight of airspace resource utilization cost in the coordination cost function	0.20	[0.15, 0.25]	Used to promote a more balanced distribution of UAVs in constrained urban airspace.

## Data Availability

Data will be made available on request.

## References

[1] Xu H., Wang L., Han W., Yang Y., Li J., Lu Y., Li J. (2023). A survey on UAV applications in smart city management: Challenges, advances, and opportunities. IEEE J. Sel. Top. Appl. Earth Obs. Remote. Sens..

[2] Mohamed N., Al-Jaroodi J., Jawhar I., Idries A., Mohammed F. (2020). Unmanned aerial vehicles applications in future smart cities. Technol. Forecast. Soc. Change.

[3] Ait Saadi A., Soukane A., Meraihi Y., Benmessaoud Gabis A., Mirjalili S., Ramdane-Cherif A. (2022). UAV Path Planning Using Optimization Approaches: A Survey: AA Saadi et al. Arch. Comput. Methods Eng..

[4] Meng W., Zhang X., Zhou L., Guo H., Hu X. (2025). Advances in UAV path planning: A comprehensive review of methods, challenges, and future directions. Drones.

[5] Zhang Z., Jiang J., Ling K.V., Wang X., Zhang W.-A. (2025). Cooperative path planning for heterogeneous UAV swarms: A Stackelberg game approach. IEEE Trans. Autom. Sci. Eng..

[6] Yan P., Ding M.-y., Zheng C.-w. (2006). Coordinated route planning via Nash equilibrium and evolutionary computation. Chin. J. Aeronaut..

[7] Yu Y., Wang H., Liu S., Guo L., Yeoh P.L., Vucetic B., Li Y. (2021). Distributed multi-agent target tracking: A Nash-combined adaptive differential evolution method for UAV systems. IEEE Trans. Veh. Technol..

[8] Su S., Ju X., Xu C., Dai Y. (2023). Collaborative motion planning based on the improved ant colony algorithm for multiple autonomous vehicles. IEEE Trans. Intell. Transp. Syst..

[9] Xu X., Xie C., Ma L., Yang L., Zhang T. (2025). Multi-objective evolutionary algorithm with two balancing mechanisms for heterogeneous UAV swarm path planning. Appl. Soft Comput..

[10] He Y., Wang M. (2024). An improved chaos sparrow search algorithm for UAV path planning. Sci. Rep..

[11] Wang Z., Sun G., Zhou K., Zhu L. (2023). A parallel particle swarm optimization and enhanced sparrow search algorithm for unmanned aerial vehicle path planning. Heliyon.

[12] Fei H., Du Z., Ma P., Liu R., Liu F., Wang M., Liu X. (2026). A Multi-Strategy Particle Swarm Optimization Algorithm for Three-Dimensional Path Planning of Amphibious Unmanned Aerial Vehicles. Eng. Appl. Artif. Intell..

[13] Merheb A.-R., Noura H., Bateman F. (2017). Emergency control of AR drone quadrotor UAV suffering a total loss of one rotor. IEEE/ASME Trans. Mechatron..

[14] Konar M. (2020). Simultaneous determination of maximum acceleration and endurance of morphing UAV with ABC algorithm-based model. Aircr. Eng. Aerosp. Technol. Int. J..

[15] Yang H., He Y., Xu Y., Zhao H. (2023). Collision avoidance for autonomous vehicles based on MPC with adaptive APF. IEEE Trans. Intell. Veh..

[16] Sun H., Dai L., Wang P., Systems E. (2024). An efficient moving obstacle avoidance scheme for UAVs via output feedback robust MPC. IEEE Trans. Aerosp. Electron. Syst..

[17] Tang H., Chen Y. (2024). Dynamic Event-Triggered Distributed MPC for Heterogeneous UAVs–UGVs Against DoS Attacks. IEEE Trans. Aerosp. Electron. Syst..

[18] Cai Z., Zhou H., Zhao J., Wu K., Wang Y. (2018). Formation Control of Multiple Unmanned Aerial Vehicles by Event-Triggered Distributed Model Predictive Control. IEEE Access.

[19] Gräfe A., Eickhoff J., Trimpe S. (2022). Event-triggered and distributed model predictive control for guaranteed collision avoidance in UAV swarms. IFAC-PapersOnLine.

[20] Redding J., Boskovic J., Mehra R., Rui C. Heterogeneous Cooperative Control of Multiple UAVs with Collaborative Assignment and Reactive Motion Planning. Proceedings of the AIAA Guidance, Navigation and Control Conference and Exhibit.

[21] Wang H., Lou S., Jing J., Wang Y., Liu W., Liu T. (2022). The EBS-A* algorithm: An improved A* algorithm for path planning. PLoS ONE.

[22] Dijkstra E.W. (1959). A note on two problems in connexion with graphs. Numer. Math..

[23] Wu W., Kong C., Xiao Z., Huang Q. (2026). 3D-MIHE-RRT-A*: Multi-indicator heuristic evaluation hybrid path planning algorithm for UAV navigation in complex environments. Measurement.

[24] Zhang Z., Wu J., Dai J., He C. (2021). Optimal path planning with modified A-Star algorithm for stealth unmanned aerial vehicles in 3D network radar environment. Proc. Inst. Mech. Eng. Part G J. Aerosp. Eng..

[25] Yoo Y.-D., Moon J.-H. (2025). Study on A-Star Algorithm-Based 3D Path Optimization Method Considering Density of Obstacles. Aerospace.

[26] Khuat T.H., Bui D.-N., Nguyen H.T.T., Trinh M.L., Nguyen M.T., Phung M.D. (2025). Multi-Goal Rapidly Exploring Random Tree with Safety and Dynamic Constraints for UAV Cooperative Path Planning. IEEE Trans. Veh. Technol..

[27] Javed S., Hassan A., Ahmad R., Ahmed W., Ahmed R., Saadat A., Guizani M. (2024). State-of-the-Art and Future Research Challenges in UAV Swarms. IEEE Internet Things J..

[28] Chen Y., Zhang D., Jing H., Huang Y., Li X. (2026). An Adaptive Multi-Population Cooperative Whale Optimization Algorithm for global optimization and 3D UAV path planning. Adv. Eng. Inform..

[29] Zhang S.W., Wang L. (2025). Multi-UAV cooperative path planning based on chaotic grey wolf optimiser. Aeronaut. J..

[30] Wu Y., Liang T., Gou J., Tao C., Wang H. (2023). Heterogeneous Mission Planning for Multiple UAV Formations via Metaheuristic Algorithms. IEEE Trans. Aerosp. Electron. Syst..

[31] Lv W., Han Z. Path Planning of UAVs Swarm Based on Wolf Pack Algorithm with Composite Walk-Fly Mechanism. Proceedings of the 2024 9th International Symposium on Computer and Information Processing Technology (ISCIPT).

[32] Qian W., Yi W., Yuan S., Guan J. (2025). Control-Oriented Real-Time Trajectory Planning for Heterogeneous UAV Formations. Drones.

[33] Lyu Z., Gao Y., Chen J., Du H., Xu J., Huang K., Kim D.I. (2026). Empowering Intelligent Low-Altitude Economy with Large AI Model Deployment. IEEE Wirel. Commun..

[34] Westheider J., Rückin J., Popović M. Multi-UAV Adaptive Path Planning Using Deep Reinforcement Learning. Proceedings of the 2023 IEEE/RSJ International Conference on Intelligent Robots and Systems (IROS).

[35] Gharehchopogh F.S., Namazi M., Ebrahimi L., Abdollahzadeh B. (2023). Advances in Sparrow Search Algorithm: A Comprehensive Survey. Arch. Comput. Methods Eng..

[36] Yang C., Yang H., Zhu D., Hu Y., Zhang Y., Ma H., Zhang D.J.C.C. (2024). A multi-mechanism balanced advanced learning sparrow search algorithm for UAV path planning. Clust. Comput..

[37] Chen D., Zhao J., Huang P., Deng X., Lu T. (2021). An improved sparrow search algorithm based on levy flight and opposition-based learning. Assem. Autom..

[38] Liu Q., Zhang Y., Li M., Zhang Z., Cao N., Shang J. (2021). Multi-UAV Path Planning Based on Fusion of Sparrow Search Algorithm and Improved Bioinspired Neural Network. IEEE Access.

[39] He Y., Wang M. (2024). Dynamic step opposition-based learning sparrow search algorithm for UAV path planning. Clust. Comput..

[40] Meyer F., Glock K., Sayah D. TOP-UAV: Open-Source Time-Optimal Trajectory Planner for Point-Masses Under Acceleration and Velocity Constraints. Proceedings of the 2023 IEEE/RSJ International Conference on Intelligent Robots and Systems (IROS).

[41] Romero A., Penicka R., Scaramuzza D. (2022). Time-Optimal Online Replanning for Agile Quadrotor Flight. IEEE Robot. Autom. Lett..

[42] Hu C., Meng Z., Qu G., Shin H.-S., Tsourdos A. (2020). Distributed Cooperative Path Planning for Tracking Ground Moving Target by Multiple Fixed-wing UAVs via DMPC-GVD in Urban Environment. Int. J. Control Autom. Syst..

[43] Liang S., Boudaoud M., Morin P., Cailliez J., Cagneau B., Rong W., Régnier S. (2021). Model Predictive Control with Obstacle Avoidance for Inertia Actuated AFM Probes Inside a Scanning Electron Microscope. IEEE Robot. Autom. Lett..

[44] Chen Y., Wang C., Zeng W., Wu Y. (2021). Horizontal nonlinear path following guidance law for a small UAV with parameter optimized by NMPC. IEEE Access.

[45] Aliyari M., Wong W.-K., Bouteraa Y., Najafinia S., Fekih A., Mobayen S. (2022). Design and implementation of a constrained model predictive control approach for unmanned aerial vehicles. IEEE Access.

[46] Abughalieh K.M., Alawneh S.G. (2019). A Survey of Parallel Implementations for Model Predictive Control. IEEE Access.

[47] Abdolhosseini M., Zhang Y.M., Rabbath C.A. (2012). An Efficient Model Predictive Control Scheme for an Unmanned Quadrotor Helicopter. J. Intell. Robot. Syst..

[48] Eskandarpour A., Sharf I. (2019). A constrained error-based MPC for path following of quadrotor with stability analysis. Nonlinear Dyn..

[49] Wang Y., Zhang T., Cai Z., Zhao J., Wu K. (2020). Multi-UAV coordination control by chaotic grey wolf optimization based distributed MPC with event-triggered strategy. Chin. J. Aeronaut..

[50] Jang D., Yoo J., Son C.Y., Kim H.J., Johansson K.H. Networked Operation of a UAV Using Gaussian Process-Based Delay Compensation and Model Predictive Control. Proceedings of the 2019 International Conference on Robotics and Automation (ICRA).

[51] Souanef T. (2023). ℒ_1_ adaptive output feedback control of small unmanned aerial vehicles. Unmanned Syst..

[52] Yu S., Chen H., Feng Y., Zhang Y., Li Y., Ebenbauer C., Chen H. (2020). Nash optimality based distributed model predictive control for vehicle platoon. IFAC-PapersOnLine.

[53] Li Y., Chen W., Fu B., Liu S., Hao L., Wu Z. (2025). A Distributed Cooperative Dynamic Target Search Method for Multi-UAV Systems in Complex Adversarial Environments. IEEE Internet Things J..

[54] Lin M., Li B., Zhou B., Cecati C. (2024). Distributed stochastic model predictive control for heterogeneous UAV swarm. EEE Trans. Ind. Electron..

[55] Kulathunga G., Hamed H., Devitt D., Klimchik A. (2022). Optimization-based trajectory tracking approach for multi-rotor aerial vehicles in unknown environments. IEEE Robot. Autom. Lett..

[56] Chen M., Rincon-Mora G.A. (2006). Accurate electrical battery model capable of predicting runtime and I-V performance. IEEE Trans. Energy Convers..

[57] Hardy C. (2014). Hysteresis. Pierre Bourdieu.

[58] Eberhart R., Kennedy J. (1995). A new optimizer using particle swarm theory. MHS’95. Proceedings of the Sixth International Symposium on Micro Machine and Human Science.

